# Reversible and long-term immobilization in a hydrogel-microbead matrix for high-resolution imaging of *Caenorhabditis elegans* and other small organisms

**DOI:** 10.1371/journal.pone.0193989

**Published:** 2018-03-06

**Authors:** Li Dong, Matteo Cornaglia, Gopalan Krishnamani, Jingwei Zhang, Laurent Mouchiroud, Thomas Lehnert, Johan Auwerx, Martin A. M. Gijs

**Affiliations:** 1 Laboratory of Microsystems, Ecole Polytechnique Fédérale de Lausanne, Lausanne, Switzerland; 2 Laboratory of Integrative Systems Physiology, Ecole Polytechnique Fédérale de Lausanne, Lausanne, Switzerland; INSERM U869, FRANCE

## Abstract

The nematode *Caenorhabditis elegans* is an important model organism for biomedical research and genetic studies relevant to human biology and disease. Such studies are often based on high-resolution imaging of dynamic biological processes in the worm body tissues, requiring well-immobilized and physiologically active animals in order to avoid movement-related artifacts and to obtain meaningful biological information. However, existing immobilization methods employ the application of either anesthetics or servere physical constraints, by using glue or specific microfluidic on-chip mechanical structures, which in some cases may strongly affect physiological processes of the animals. Here, we immobilize *C*. *elegans* nematodes by taking advantage of a biocompatible and temperature-responsive hydrogel-microbead matrix. Our gel-based immobilization technique does not require a specific chip design and enables fast and reversible immobilization, thereby allowing successive imaging of the same single worm or of small worm populations at all development stages for several days. We successfully demonstrated the applicability of this method in challenging worm imaging contexts, in particular by applying it for high-resolution confocal imaging of the mitochondrial morphology in worm body wall muscle cells and for the long-term quantification of number and size of specific protein aggregates in different *C*. *elegans* neurodegenerative disease models. Our approach was also suitable for immobilizing other small organisms, such as the larvae of the fruit fly *Drosophila melanogaster* and the unicellular parasite *Trypanosoma brucei*. We anticipate that this versatile technique will significantly simplify biological assay-based longitudinal studies and long-term observation of small model organisms.

## Introduction

Approximately 40% of the about 20,000 *C*. *elegans* protein coding genes are functional orthologues of their human counterparts [1]. Additionally, a large number of orthologues of human disease genes and disease pathways has been found, which is a major reason why *C*. *elegans* has become an attractive model organism for the study of human diseases, aging and for drug discovery [2–6]. For instance, in *C*. *elegans* models for neurodegenerative diseases, such as Alzheimer’s, Parkinson’s or Huntington’s disease, disease progression can be assessed by monitoring cluster formation of specific proteins in the worm’s body tissue [7]. Protein aggregation is a common hallmark of most neurodegenerative diseases. Furthermore, in *C*. *elegans*, as in humans, a decline in mitochondrial function plays a key role in the aging process, while altered mitochondrial dynamics is known to be implicated in many diseases, including neurodegenerative disorders [8–11]. Inhibition of mitochondrial division, for instance, attenuates disease-associated phenotypes in multiple models of neurodegenerative disease [11,12]. Mitochondrial fission and fusion therefore represent a potential therapeutic target for neurodegeneration and needs to be further explored in relevant animal models, in particular *C*. *elegans*.

Such studies mainly rely on high-resolution imaging, *e*.*g*. based on fluorescent confocal microscopy imaging, and on longitudinal observations of dynamic alterations of mitochondrial networks or cluster morphologies. High-resolution imaging over time spans of minutes up to several hours, however, requires secure immobilization of worms. Any residual agitation may blur details of tissue fine structures or of dynamic molecular events, and thus reduce the information content and quality of the assay. Moreover, long-term observation and immobilization should be carried out under physiological conditions with minimal impact on the worms’ health. Conventional immobilization methods rely on anesthetic drugs [13], such as sodium azide, phenoxypropanol, and tetramisole, to paralyze the worms, or on mechanical immobilization with glue [14]. However, anesthetics chemically disrupt normal physiological functions of the worms (*e*.*g*. sodium azide perturbs cellular activity [15]). Both types of immobilization methods inhibit the natural worm development, and worms can usually not be recovered after imaging.

As an alternative to overcome these limitations, microfluidic approaches for on-chip worm manipulation have been developed in recent years [16–18]. Different on-chip worm immobilization techniques, in particular methods based on mechanical forces, have been proposed, such as arrays of tapered channels, *e*.*g*. for performing lifelong observation [19], neuronal ablation [20] or electrophysiology experiments [21], and pressure-controlled deflectable membranes, *e*.*g*. for *in vivo* imaging of dynamic cellular processes [22] or neuronal transport [23], laser axotomy [24] and chemosensing assays [25].

Another class of devices takes advantage of Pluronic F127 (PF127), a biocompatible triblock copolymer that, in aqueous solution, undergoes thermogelling at a specific gelation temperature, *i*.*e*. a reversible sol-to-gel transition upon heating by forming a three-dimensional packing of micelles due to amphiphilic molecule interactions [26,27]. Gelation temperature and viscosity of the polymer solution depend on the PF127 concentration. Melentijevic *et al*. presented a hybrid immobilization method by placing *C*. *elegans* worms in a drop of cooled PF127 (36% w/v) + 1 mM tetramisole solution in between two coverslips, followed by gelification at room temperature, for imaging of two worm touch neurons in the head region (ASE and ALM) and mitochondria [28]. Depending on the dose of the anesthetics used, the residual motion of the head region might still occur. Additional embedding of the worms in Pluronic gel, may result in more secure immobilization conditions as demonstrated in this paper. However, as this method still involves anesthetics, adverse physiological effects, as mentioned above [15], may not be fully excluded. PF127 has also been implemented in different microfluidic devices, to trigger or improve worm immobilization through a temperature-induced sol-gel transition, *e*.*g*. in worm culture chambers [29] or trapping channels [30], in droplet-based devices [31] or in a temperature-controlled microfluidic platform for monitoring *in vivo* protein aggregation in *C*. *elegans* models [32]. Chuang *et al*. developed an optoelectric device based on the simultaneous application of laser irradiation and an electric field to enable selective worm immobilization [33]. The technique was used to track muscular degeneration during aging in a specific transgenic strain. Huang *et al*. proposed a photothermal immobilization method based on optical control of the sol-gel transition of the thermosensitive polymer via a photo-absorbing layer on the substrate [34]. This simple method, however, required specific bright field illumination conditions to trigger a sol-gel transition locally, and white light exposure had to be maintained during fluorescent imaging. For these reasons, the technique seems not easily adaptable to high-resolution confocal imaging sessions. Moreover, repeated and relatively long exposure to light stimuli is known to evoke negative phototaxis behavior in *C*. *elegans* [35].

All methods or devices discussed so far have specific drawbacks, *e*.*g*. an unknown or adverse effect on the organism’s health, the complexity of the fabrication process, a certain lack of versatility, or the requirement of technical resources that may not be available in standard biomedical research labs. Additionally, major drawbacks of PF127-based microfluidic devices are related to problems with injecting and on-chip manipulation of the viscous solution/gel and the integration of a chip temperature control system for reversible PF127 thermogelling. Hence, there is a lack of a simple approach that combines the possibility of long-term or periodic high-resolution imaging of a small model organism throughout its lifespan.

In this paper, we propose a new versatile method using a hydrogel-microbead matrix for fast and reliable, yet reversible immobilization, in particular of *C*. *elegans* at all development stages, as well as other small organisms (*D*. *melanogaster* larvae and *T*. *brucei*). The immobilization principle is based on the combined action of hydrogel in its highly viscous state and moderate compression of the worms, which is well-controlled through the microbead spacers. Our approach does not rely on a specific chip design and requires only minimal manual operation. As a proof-of-concept, we employed this simple technique for *in vivo* confocal fluorescent high-resolution imaging of mitochondrial networks in the body wall muscle cells of *C*. *elegans*. We demonstrated that our approach is suitable for the accurate observation of the long-term dynamics of mitochondrial fusion and fission processes in worms subjected to different RNA interference (RNAi) treatments. Moreover, we analyzed protein aggregate progression in *C*. *elegans* neurodegenerative models in a quantitative manner.

## Experimental

### Materials

Polystyrene microbeads with diameters of 15 μm, 30 μm and 40 μm (std. dev. <0.5 μm, coeff. var. <2%) were purchased from Sigma-Aldrich (Buchs, Switzerland). Standard glass slides (76 mm×25 mm×1 mm) and coverslips (22 mm×22 mm×0.17 mm) were obtained from Carl Roth GmbH (Arlesheim, Switzerland). Pluronic (PF127) and tetramisole hydrochloride were acquired from Sigma-Aldrich. Lysogeny broth (LB) bacterial culture medium was prepared by adding 10 g Bacto^TM^ tryptone, 5 g Bacto^TM^ yeast and 5 g NaCl in 1 L of DI H_2_O. Tetracycline and ampicillin were purchased from Sigma-Aldrich and Eurobio (Les Ulis, France), respectively. S Medium was prepared by adding 10 ml of 1 M potassium citrate pH 6, 10 ml of trace metals solution, 3 ml 1 M CaCl_2_ and 3 ml 1 M MgSO_4_ in 1 L of S Basal. S Basal, LB and S Medium were sterilized by autoclaving. All chemicals used in S Basal, LB and S Medium solutions were purchased from Sigma-Aldrich. The fly medium was prepared with 6.2 g agar powder (ACROS No. 400400050), 58.8 g Farigel wheat (Westhove N. FMZH1), 58.8 g yeast (Springaline BA10), 100 mL grape juice, 4.9 mL propionic acid (Sigma No. P1386), 26.5 mL of methyl 4-hydroxybenzoate (VWR No. ALFAA14289.0) solution (400 g/L) in 95% ethanol and 1 L water. The modified HMI-9 medium (for 1 L water) was prepared using 17.66 g IMDM (Life Technologies Cat # 12440–046), 3.024 g NaHCO_3_, 28.2 mg bathocuproine in 10 mL water, 39.0 mg thymidin in 10 mL water, 14.0 μL 2-mercaptoetanol in 10 mL water, 1.0 mg Pen Strep in 10 mL water, 136.0 mg hypoxanthine in 20 mL water, 100 mL FBS, 182.0 mg cysteine in 10 mL water. All chemicals were purchased from Sigma-Aldrich.

### Bacterial food source

Bacterial culture of *Escherichia coli* OP50 was obtained by inoculating a small amount of OP50 glycerol stock in LB medium without antibiotic. The bacteria were grown at 37°C overnight in an orbital shaker and kept at 4°C. Bacterial culture of the HT115 bacteria strain was done in two steps. For the first culture, bacteria were grown at 37°C overnight in an orbital shaker in LB medium with tetracycline (0.0125 mg/ml final concentration) and ampicillin (0.1 mg/ml final concentration). The following day, for the second culture, they were allowed to grow for 8 hours in LB medium with only ampicillin at the same concentration as before. These bacterial solutions were used to seed nematode growth medium (NGM) agar plates on the same day. The plates were left to dry overnight before worm transfer.

### Worm and other small organism culture

*C*. *elegans* strains used were wild-type Bristol N2, SJ4103 (zcIs14 [*myo-3*::GFP(mit)]), AM140 (rmIs132[*unc-54p*::Q35::YFP]), NL5901 (pkIs2386[*unc-54p*::alphasynuclein::YFP + *unc-119*(+)]) and AM725 (rmIs290[*unc-54p*::Hsa-sod-1(127X)::YFP]. All strains were provided by the Caenorhabditis Genetics Center (University of Minnesota). *C*. *elegans* strains were cultured at 20°C on NGM plates seeded with *E*. *coli* OP50 as a food source. Bacteria cultures were grown overnight at 37°C. Silencing of *fzo-1* and *drp-1* genes were obtained using the RNAi technique by feeding worms with bacteria expressing dedicated double-stranded RNA [Kamath, 2001]. *fzo-1* (ZK1248.14), *drp-1* (T12E12.4) and *E*. *coli* HT115 (empty vector) clones were obtained from the Ahringer *C*. *elegans* RNAi feeding library.

*D*. *melanogaster* wild-type (Canton-S) lines were obtained from the Laboratory of Systems Biology and Genetics (EPFL) and reared at room temperature on a standard fly medium. *T*. *brucei* MiTat 1.2 trypanosomes were grown in HMI-9 cell medium at 37°C at 5% CO_2_. Populations were kept below 10^6^ cells per mL by repetitive splitting. After a maximum of 15 splittings, trypanosomes were discarded and fresh populations were defrosted.

### Image acquisition and processing

An inverted microscope (Axio Observer, Zeiss) equipped with two illumination systems was used: (i) a pE-100 white LED illumination system (CoolLED, Andover, UK) for brightfield imaging, and (ii) a Lambda DG-4 illumination system (Sutter Instruments, Novato, CA, USA) for fluorescence imaging. The microscope had a motorized xy-stage and the imaging process was controlled using VisiView Premier Image acquisition software (Visitron, Puchheim, Germany). Images were acquired through a Hamamatsu Orca-ER CCD camera (Hamamatsu, Japan). Image processing was performed with Fiji (http://imagej.nih.gov/ij; version 1.47b). In order to quantify different aggregate parameters, images were first converted into 8-bit format, followed by the application of a radius 2 median filter and a suitable signal threshold. Finally, the FIJI particle analyzer plugin was used in order to quantify the number of aggregates, size and total area. For our fluorescence imaging experiments, typical exposure times were in the range of 100–150 ms, however sharp images of cellular structures can be obtained for exposure times up to 500 ms. Time-lapse sequences were recorded with 5 s intervals up to 3 hours. Our immobilization method is also compatible with recording of high resolution z-stacks with fluorescence or confocal techniques, respectively.

### Confocal imaging of mitochondrial morphology

For confocal imaging, a *C*. *elegans* strain expressing mitochondrial-targeted Green Fluorescent Protein (GFP) driven by the muscle-specific *myo-3* promoter (SJ4103) was used. Worms were immobilized using the gel-based technique detailed in this paper or with 6 mM solution of tetramisole hydrochloride (control group) in S Medium and mounted on 6% agarose pads on glass slides. Images of worms were acquired using a Zeiss LSM 700 upright confocal microscope (Carl Zeiss AG, Oberkochen, Germany, 63× NA 1.4) under non-saturating exposure conditions. All pictures of mitochondrial morphology were taken in body wall muscles from the upper or lower part of the worm, excluding the regions of oesophagus and vulva.

### Statistical analysis

One-way analysis of variance was used in comparisons of 3 or more groups. Student t test was used for comparison of 2 groups. All p values were 2-sided, and P < 0.05 was considered statistically significant. The analysis was conducted using GraphPad-Prism4 software.

### Gel-based immobilization protocol

**Fig 1:**
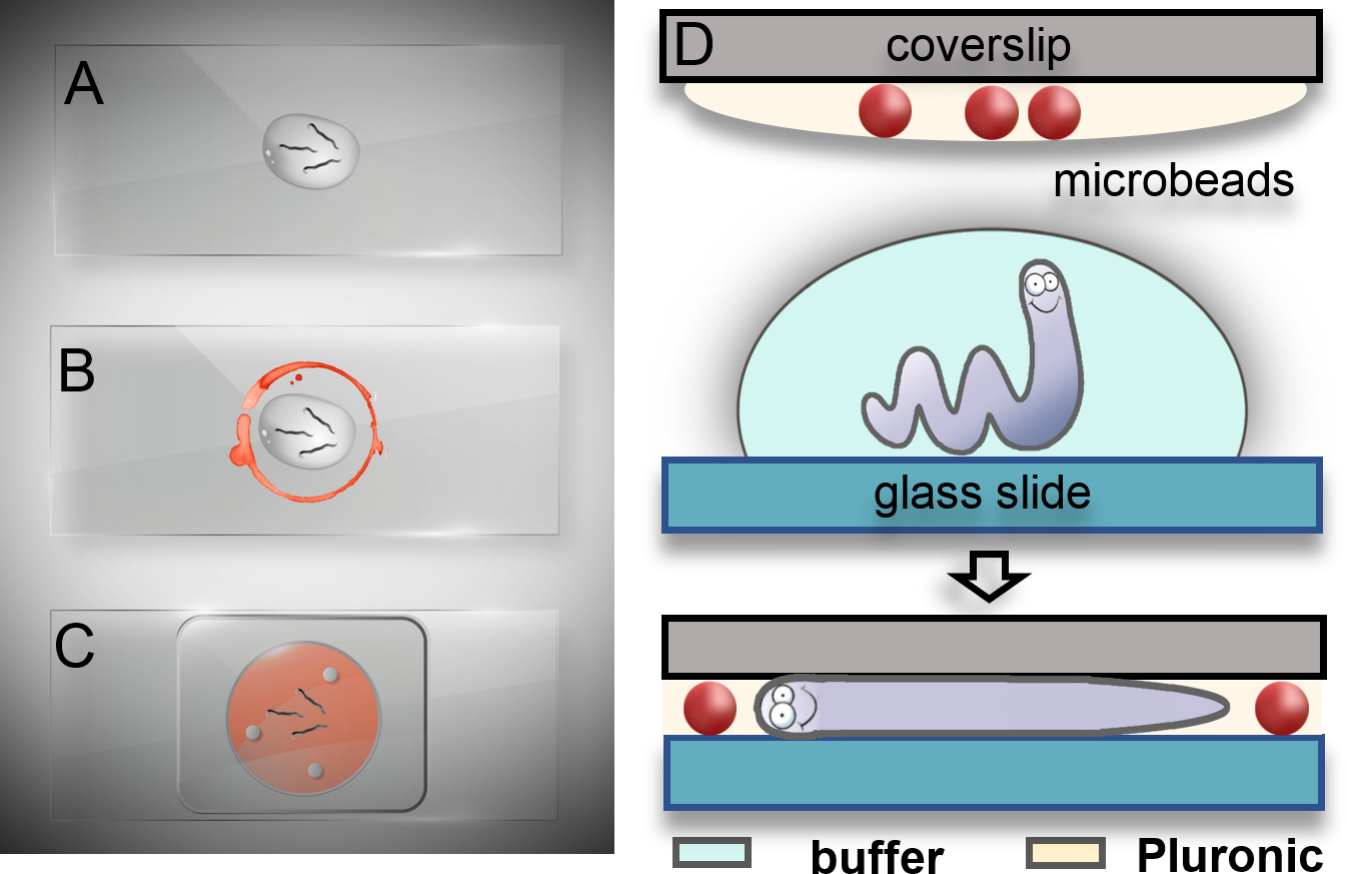
*C*. *elegans* immobilization protocol using a hydrogel-microbead matrix. (A) Worms are transferred from an agar plate into a droplet of liquid S Medium on a glass slide. (B) Precooled (~4°C) liquid Pluronic-microbead suspension is applied on the glass slide around the S Medium (red trace) and on a coverslip (not shown here). (C) The coverslip is positioned upside down and worms are immobilized in the gel-microbead matrix (red) in between the two glass substrates after thermalization (*T* ≈ 25°C). (D) Schematics of the worm immobilization technique with microbead spacers.

For immobilization experiments, PF127 in water (30% w/v) was used, either mixed with polystyrene microbeads (for *C*. *elegans* adult worms) or as pure hydrogel (for *C*. *elegans* larvae, *D*. *melanogaster* larvae and *T*. *brucei*). To obtain a low viscosity Pluronic solution (liquid state), a 1 ml syringe filled with PF127 was stored at 4°C before and after each experiment. The worm immobilization protocol comprises the steps depicted in Fig 1. First, a 10 μl droplet of S Medium was pipetted on a glass slide to provide a suitable liquid environment for the worms. About 10 worms were then transferred from an NGM agar plate into the droplet by using a worm pick (Fig 1A). In the next step, the precooled low-viscous PF127 solution or PF127-microbead suspension was applied around the S Medium droplet on the glass slide (Fig 1B) and on a coverslip, which was placed upside down over the worms (Fig 1C). Thereby S Medium was expelled from the center area, leaving the worms in the gel matrix. Gelation prior to imaging was obtained by thermalization of the PF127 solution in between the glass slides at room temperature. For this reason, manipulation at room temperature had to be done reasonably fast (≤ 2 min).

The principle of adult worm confinement and immobilization is schematically shown in Fig 1D. Applying a uniform pressure onto the coverslip results in an evenly distributed monolayer of beads and worms trapped within the gel matrix. The presence of microbeads with suitable diameter (30 μm or 40 μm in our experiments) between the two glass surfaces prevents damage to the worms, thereby allowing gentle and well-controlled compression of the worms’ body.

### Worm recovery

For worm recovery after gel immobilization, worms were released by carefully lifting the coverslip. The hydrogel then formed a dendritic structure on the surface of the glass slide, where worms could be easily found. Worms were set free by rapid dissolution of the surrounding gel in a small drop of S Medium. Then they could be easily placed back on an agar plate by pipetting the liquid. For worm recovery after using 1mM tetramisole, worms were put onto a drop of S Medium (5–10 μl) to wash out the anesthetic and transferred to an agar plate using a worm pick.

### Motility and fertility assay

For motility, worms recovered from the chip or tetramisole either after short-term (10 min) or long-term immobilization (60 min) were first transferred to S Medium in a glass well and observed for measuring the thrashing frequency using a stereomicroscope during 30 s intervals. Subsequently, each worm was transferred back to the agar plate for normal culture. These measurements were done 10 min, 3 h and 1 day after release.

For fertility, 10 individual adult worms, after being exposed to different immobilization conditions (1 h and 2 h in PF127, 1h tetramisole, and control), were transferred onto separate NGM plates and cultured at 20°C. After day 1, 2 and 3 worms were again transferred on a fresh plate and progeny was left to develop for 3 days before counting.

## Results and discussion

### Thermogelling of aqueous Pluronic solutions

**Fig 2:**
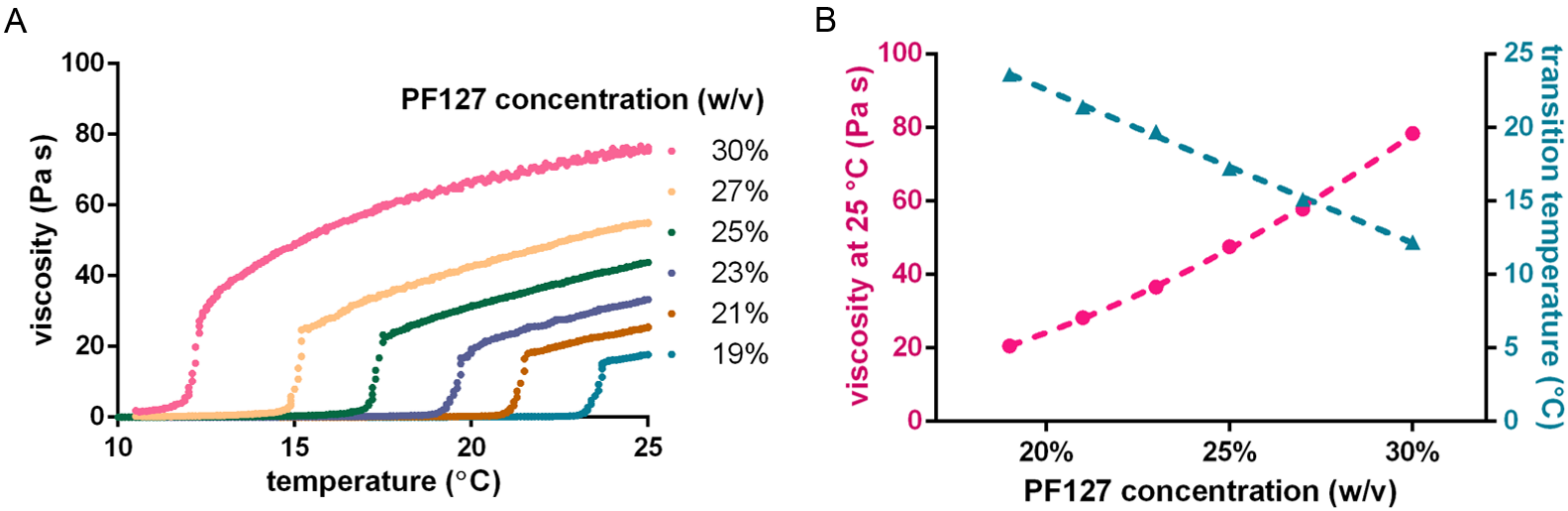
Characterization of different Pluronic (PF127) solutions. (A) PF127 viscosity as a function of temperature for a range of concentrations, measured with a cone-plate viscometer. The viscosity curves show a sharp rise at a specific temperature, corresponding to the sol-gel transition. (B) The viscosity at 25°C follows a quadratic progression as a function of PF127 concentration. The sol-gel transition temperature decreases linearly with increasing Pluronic concentration.

The accessible parameter range for worm immobilization was first assessed via viscosity measurements of PF127 solutions of different concentrations and over the temperature range of interest (Fig 2A). The PF127 viscosity was measured by a cone/plate viscometer (*Bohlin C-VOR Shear Rheometer*) at constant shear rate. The temperature of the polymer solution was controlled via a water jacket system. The sol-gel transition temperature showed a linear drop with increasing PF127 concentration and the viscosity reached room temperature (25°C) depends quadratically on the polymer concentration (Fig 2B). 30% w/v of PF127 in water is the highest concentration that can conveniently be prepared at room temperature. For our experiments, we used this concentration, as it has the highest viscosity in the gel phase and a convenient sol-gel transition temperature of 12.0±0.5°C.

### Immobilization of *C*. *elegans* larvae

Larvae are incorporated in a pure droplet of PF127 solution (no microbeads) which is sandwiched in between the two glass slides. In this case, the coverslip is only gently pressed from the top to flatten the Pluronic droplet in order to loosely align the larvae in the focal plane for imaging. The spacing is comparable or slightly larger than the body diameter, thus no mechanical compression of the larvae themselves occurred, nor was required for full immobilization. Using PF127 (30% w/v), viscoelastic forces at room temperature (25°C) within the gel matrix were sufficient for full immobilization of *C*. *elegans* larvae at all stages from L1 to L4 (body length in the range from ~250 μm to ~650 μm [36], diameter from ~10 μm to ~25 μm [37], respectively).

### Adult worm immobilization using a gel-microbead matrix

**Fig 3:**
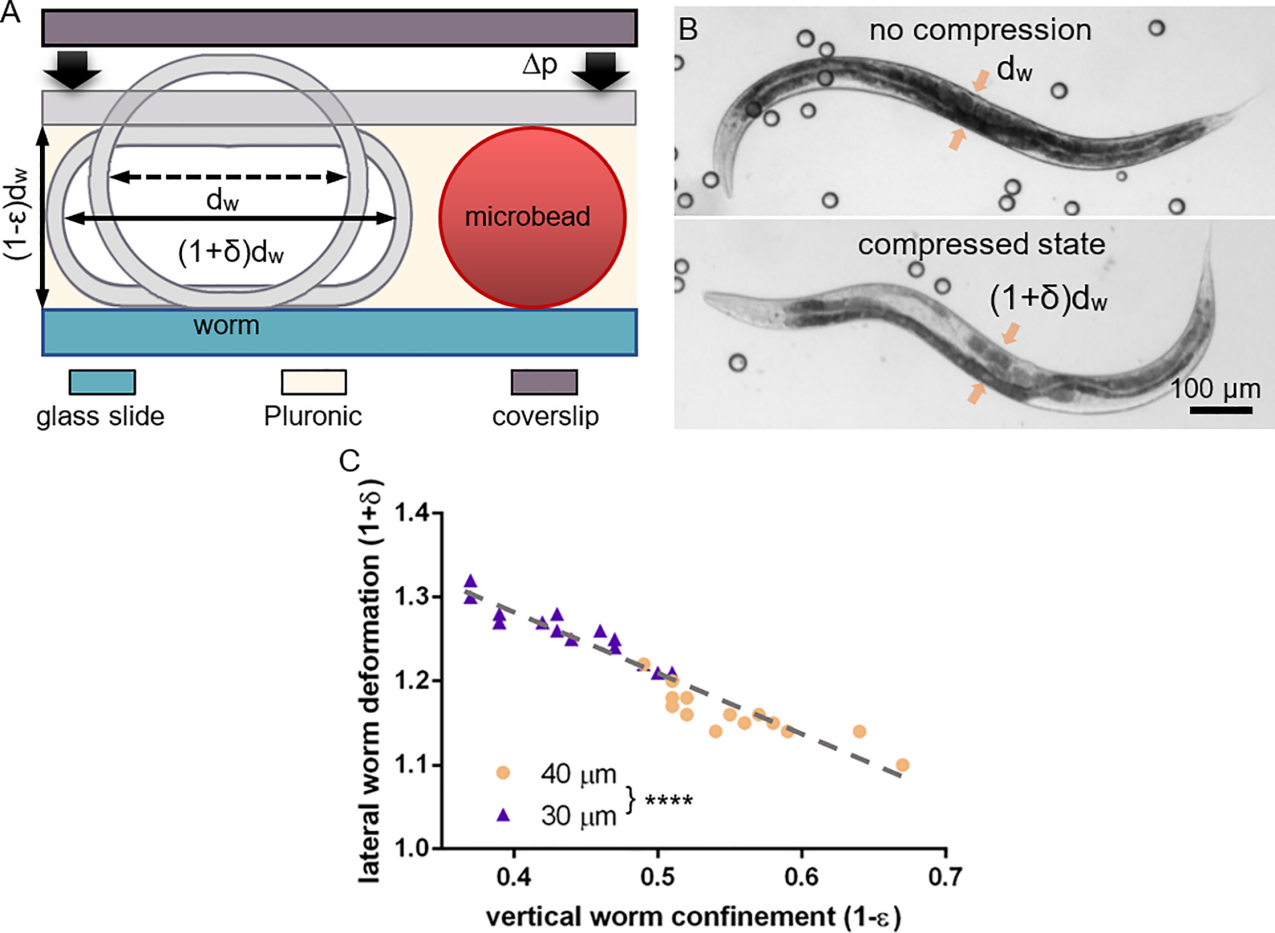
The mechanism of *C*. *elegans* immobilization in the hydrogel-microbead matrix. (A) Application of compression in the normal direction increases friction between the worm body and the glass surfaces. (1-ε) and (1+δ) denote the vertical and lateral deformation of a worm with a body diameter *d*_*w*_. (B) Bright field images of an adult hermaphrodite worm in a hydrogel-microbead matrix on a glass slide: freely moving (without coverslip), and in a compressed state with a coverslip. The vertical confinement is defined by the bead diameter. Arrows indicate the location where the lateral dorsoventral deformation of the worm body was measured. (C) Worm body extension (1+δ as a function of the degree of compression/confinement (1-ε for two different microbead spacers (40 μm and 30 μm). The range of values is determined by body diameter variations of the unsynchronized worm population used here. (**** p≤0.001, n = 15 for each group).

Worms have strong somatic muscles for locomotion based on lateral undulatory motion patterns. Thus, accurate immobilization with negligible residual motion for long-term imaging merely based on viscoelastic forces, as for larvae, is not feasible. To immobilize adult *C*. *elegans*, we added microbeads to the PF127 gel matrix that function as spacers between the two glass slides, providing a well-defined vertical confinement of the worms. Fig 3A depicts this principle in detail. In the uncompressed state, viscous forces on the worm body dominate, and significantly slow down the worms’ motion. But even with the highly viscous Pluronic 30% w/v gel at 25°C, such forces are not sufficient to fully inhibit motion of adult worms. Slight compression of a worm in between the two glass interfaces, however, increases friction forces between the worm body and the adjacent glass substrates. If the combined effect of friction and viscoelastic gel forces exceeds the worms’ body forces, worms’ motion may be inhibited.

To observe physiological processes, and possibly to recover the worms after imaging, the worm body has to be compressed to a level that is not harmful. In order to choose the adequate size for the microbeads, we first evaluated the degree of compression of the worm body for different bead diameters. For this, we assume that a worm with a circular cross-section *d*_*w*_ is subjected to a vertical confinement (1-ε)·*d*_*w*_ in between the two glass slides, which causes a lateral extension with a dorsoventral width (1+δ)·*d*_*w*_ at mid-body location (Fig 3A). Typical values for *d*_*w*_ are the range of 60 to 80 μm, increasing slightly from the young adult to the old adult stage. Representative bright field images of an adult worm are shown in Fig 3B, in the uncompressed and in the compressed state, respectively. To evaluate the maximum pressure that adult worms can bear, we tested 40 μm, 30 μm and 15 μm microbeads as spacers, corresponding to increasing amounts of vertical and lateral deformation of the worms. For that, worms were first placed in a microbead-medium drop (5–10 μl, 2.5% by volume) on a glass slide, and then pressure was applied from the top with a second coverslip. The worm diameters were measured twice before and after compression, using a digital microscope (Keyences VHX-700F). According to our study, worms can safely withstand deformations limited by 40 μm (ε≈0.33–0.52 and δ≈0.10–0.22) and 30 μm microbeads (ε≈0.50–0.63 and δ≈0.21–0.30), respectively. However, with 15 μm beads compression was too strong (ε≈0.75), causing irreversible physical damage to the worms (explosion). For this evaluation, worm populations have not been age-synchronized, thus the range of ε and δ values is determined by the variation of worm body diameters in the adulthood stage. In Fig 3C we plotted experimental values for the lateral worm body deformation (1+δ) as a function of the vertical confinement (1-ε).

Our method differs from another bead-based technique proposed by Kim *et al.[38]*. These authors evaluated the immobilization of worms placed on agarose pads mixed with nanoparticles (diameters 0.05 μm, 0.1 μm, 0.2 μm or 0.5 μm) and hypothesized that the large surface area of small diameter beads may play a role in increasing both contact area and the interfacial shear strength with the agarose matrix, thereby increasing friction. In this case, the spacing of the glass slides is larger than the worms’ diameter, nevertheless the worm body dorsoventral width increased up to 40% (for 10% agarose in NGM buffer) by compression due to the stiffness of the agarose. An inconvenience of this approach is that agarose pads do not provide the versatility of repeated PF127 thermogelling. Furthermore, adult worms can swallow beads up to a diameter of a few micrometer [39], thus using nanobeads that can be ingested might have an adverse effect on the worm’s physiological condition.

### Quantification of worm motility in different media

**Fig 4:**
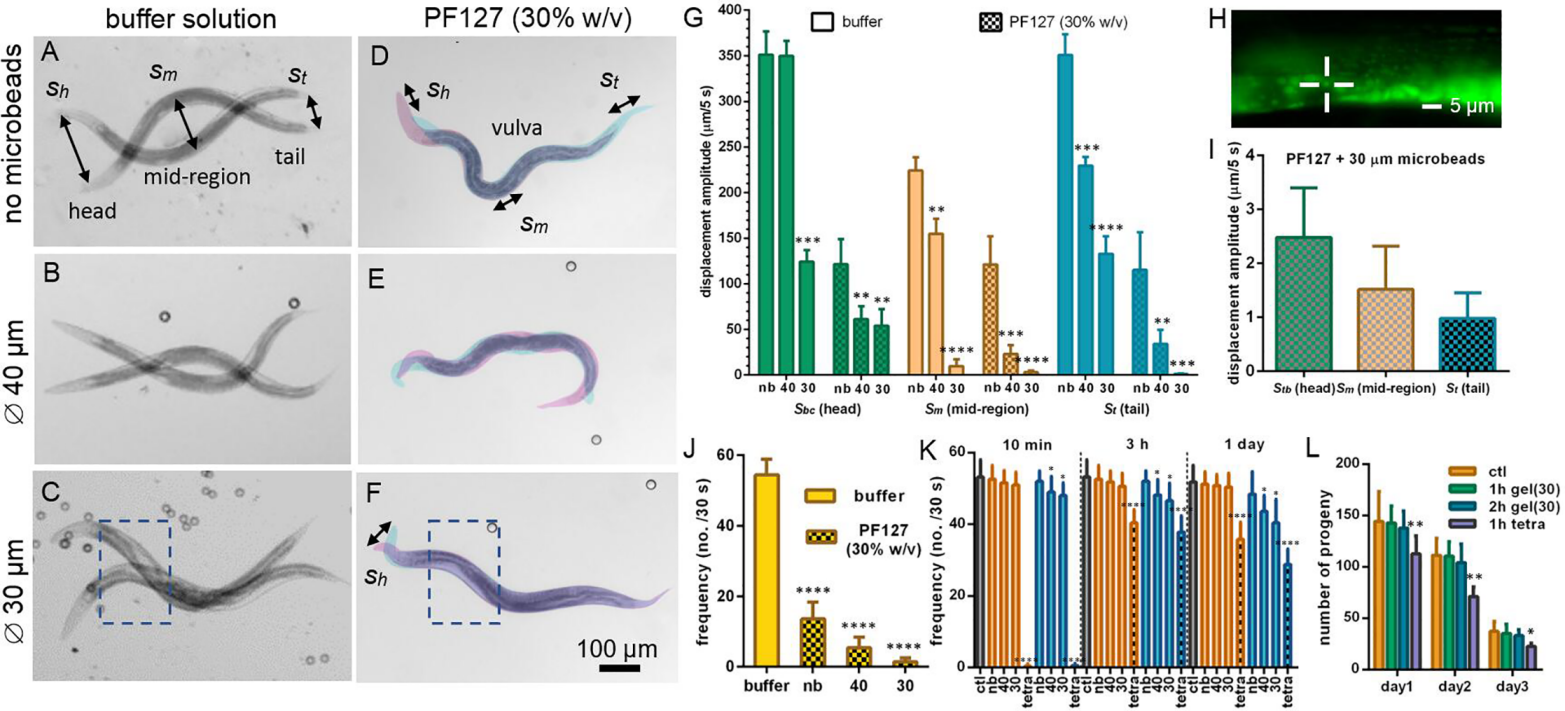
Evaluation of *C*. *elegans* immobilization and recovery for different conditions. (A-F) Pairs of successive bright field images (10× objective) give an indication of adult worm motility under different conditions: (A-C) in buffer solution (one stroke intervals) and (D-F) in a PF127 (30% w/v) hydrogel matrix (using false colors to visualize two successive snapshots (5 s intervals). 3 different degrees of worm compression have been applied in either case: (A, D) no compression (no microbeads), and vertical confinement defined by 40 μm (B, E) or 30 μm (C, F) spacer microbeads, respectively. Rectangles indicate regions of interest for high-resolution imaging. Displacement amplitudes of 3 distinct worm body regions (*s*_*bc*_ head close to the buccal cavity, *s*_*m*_ mid-region and *s*_*t*_ tail), as indicated in (A) or (D), were measured. (G) Mean amplitude of displacement over 5 s intervals of the 3 worm body regions, in buffer solution or in PF127 under different conditions: no beads/no compression (nb), and with 40 μm or 30 μm spacer beads. Data is presented as mean±SD, ** p ≤ 0.01, *** p ≤ 0.001 and **** p ≤ 0.0001 *vs*. nb in each condition, n = 15 for each group. (H) Fluorescent image of mitochondria aggregates in a *Pmyo-3*::*mito*::*GFP* worm immobilized in a PF127-microbead (30 μm) matrix. A selected mitochondrial structure in mid-body region close to vulva is indicated by crosshairs (63× objective). (I) Mean absolute displacement over 5 s intervals of different selected mitochondrial structures near terminal bulb (*s*_*tb*_), vulva in the mid-body region (*s*_*m*_) and anus (*s*_*t*_). Animals were immobilized in a PF127-microbead (30 μm) matrix. Data is presented as mean±SD, n = 10 for each group. (J) Frequency of repeating motion patterns for freely moving adult worms (thrashes in buffer) and during immobilization under different conditions in PF127 (mainly forward-backward motion). Data is presented as mean±SD, **** p≤ 0.0001 *vs* buffer group, n = 15 for each group. (K) Evaluation of worm recovery from short-term (10 min, orange bars) and long-term (1 hour, blue bars) immobilization in PF127 (30% w/v) and tetramisole (1mM). The thrashing frequency was measured 10 min, 3 h and 1 day after release, respectively. The control population was maintained in buffer solution (no immobilization) all the time (ctl, grey bar). Data is presented as mean±SD; for short-term immobilization, ***p≤ 0.001 *vs* ctl group; for long-term immobilization, *p≤ 0.05 and ****p≤ 0.0001 *vs* ctl group. N = 15 for each group. (L) Evaluation of worm fertility (N2 wild type) after long-term immobilization (1 hour and 2 hours) in PF127 (30% w/v) and using tetramisole (1mM, 1 hour) at 20°C. Measurements were made at adult stage at day 1, 2 or 3 after release. Data is presented as mean±SD, *p≤ 0.05 and **p≤ 0.01 *vs* ctl group of each day, n = 10 for each group.

In order to evaluate more accurately the importance of the hydrogel-microbead matrix for worm immobilization, we applied different degrees of compression (no compression, *i*.*e*. without beads and a loosely positioned coverslip for aligning worms in the focal plane, and compression limited by 40 μm or 30 μm spacer microbeads). We compared the impact on worm motility in buffer solution or in PF127 (30% w/v) gel, respectively. Fig 4A–4C shows two superimposed bright field images of a worm’s position in buffer solution for the three configurations. This image series, exhibiting the typical wave-like motion of worms swimming in aqueous solution, reveals stronger restriction of motion with increasing confinement of the worms, and a transition from C-shape (free motion) to more S-shape patterns (due to stronger worm confinement and friction in the thicker mid-body region). Likewise, Fig 4D–4F show snapshots of worms in PF127 for the different compressive states. In the gel matrix, the worm motion patterns are dramatically different. Due to high viscoelastic forces, the worms can hardly perform lateral undulations but exhibit mainly forward-backward locomotion, similar to their characteristic crawling behavior on agar plates. Displacement amplitudes are considerably smaller than in buffer solution, and we used false colors to highlight two extreme worm positions. As emphasized visually in Fig 4F, using 30 μm spacer beads in the gel matrix, an adult worm can be immobilized nearly completely over the full body length (except a small section of the head). Dashed rectangles in Fig 4C and 4F indicate the regions of interest for the bio-assays and imaging experiments performed in the frame of this work. Comparing both pictures clearly reveals significantly improved and apparently complete immobilization of this portion of the worm body in the PF127-microbead matrix.

To assess the worms’ motility quantitatively, we measured the mean displacement amplitudes of three distinct body regions, *i*.*e*. of the head close to the buccal cavity (*s*_*bc*_), the mid-body region (*s*_*m*_) and the tail (*s*_*t*_), corresponding to oscillation amplitudes in buffer solution or to the amplitudes of longitudinal displacement in the gel matrix, respectively (as indicated in Fig 4A and 4D). Mean absolute displacement values over 5 s intervals were determined by using a digital microscope (Keyences VHX-700F) and imageJ. The results are summarized in Fig 4G. In buffer solution, the thrashing amplitude *s*_*m*_ without compression in the mid-body region is 225±15 μm, whereas *s*_*bc*_ and *s*_*t*_ reach values up to 350±30 μm. Upon compression using 30 μm beads, *s*_*m*_ decreases to 12±3 μm. Head and tail regions, having smaller diameters than the mid-body region, still show strong undulation in this case, with amplitudes in the range of 120–130 μm. In PF127 (30% w/v) gel at room temperature, overall displacement amplitudes in all three worm body regions were significantly reduced. A mid-body amplitude *s*_*m*_ of 120±30 μm was measured without compression. Using 40 μm beads in PF127 gel, *s*_*m*_ decreased to 24±6 μm, a value that still might be too large for most high-resolution imaging protocols. Upon further compression with 30 μm spacer beads, however, the stroke amplitude *s*_*m*_ of the mid-body and *s*_*t*_ of the tail region fell to values in the range of the accuracy of measurement (20× objective, *s*_*m*_ = 1.6±0.8 μm and *s*_*t*_ = 1.0±0.4 μm, respectively). This configuration was suitable to perform the high-resolution wide-field and confocal imaging experiments described below. A small part of the head region (*i*.*e*. an about 50 μm long portion from the buccal cavity) is only weakly compressed due to its conical shape and still wiggles continuously (*s*_*bc*_ = 55±15 μm). However, as emphasized by the corresponding false-color image shown in Fig 4F, this agitation is not transmitted to the worm body, in particular to regions where imaging of organs or cells would take place. It is worth mentioning, that even in the hypothetic case of perfect immobilization of the worm’s cuticle, the residual motion of inner organs or body wall muscles, exhibiting pulsating displacements in the range of a few μm, imposes a natural limit to high-resolution imaging of living physiologically active worms.

To quantify the degree of immobilization at cellular level, we tracked the movement of mitochondrial structures in a *Pmyo-3*::*mito*::*GFP* worm immobilized in a PF127-microbead (30 μm) matrix. Mitochondrial displacements in the head near the terminal bulb, *i*.*e*. about 100 μm away from the buccal cavity (*s*_*tb*_, S1 Movie), in the mid-body region (*s*_*m*_, near vulva, S2 Movie) and in the tail (*s*_*t*_, near anus), were recorded at high-resolution (63× objective) over a period of 30 min (5 s intervals), respectively. We measured the coordinates of the selected mitochondrial structures (*e*.*g*. as indicated by crosshairs in Fig 4H) in each frame and calculated the average displacement of the structures between consecutive images. The results for each region are plotted in Fig 4I. The mitochondrial structures were carefully selected from non-fusion/fission individuals, which might cause measurement deviation. We found that displacement amplitudes at cellular level in these three distinct body regions, in particular the motions of the anterior and terminal bulb region, which are also regions of interest for neuronal imaging, exhibit only few μm displacements over the 5 sec intervals. This appears to be largely due to residual motion.

The imaging resolution is also affected to a certain extent by the speed or characteristic time scale of the displacement, which should be compared to the exposure time required for the experiment. For very short exposure times in the ms-range, full-worm scale images could be even obtained from worms moving freely in aqueous solution if high-speed sensitive cameras are used. However, tracking a worm in a population or localizing specific features inside a worm would not be possible in this case. Much longer exposure times (hundreds of ms) are necessary for low-intensity signals and/or high magnification imaging, thus requiring adequate worm immobilization. In this regard, we determined the thrashing frequency for freely moving worms in buffer solution and during the different immobilization conditions. Fig 4J summarizes these values, showing that also this parameter decreases drastically with increasing compression.

### Worm recovery after immobilization

Worm recovery and possible adverse effects of the gel-based technique on worm physiology were assessed by measuring the worms’ motility and fertility. In Fig 4K, values of the thrashing frequency after recovery from short-term (10 min, orange bars) or long-term immobilization (60 min, blue bars) are plotted and compared to a control population that was maintained in buffer solution (no immobilization) and tetramisole-treated (1mM) populations. These values were assessed 10 min, 3 h and 1 day after release. Typically, for standard imaging techniques, immobilization durations no longer than a few minutes are required. For instance, with the fluorescence wide-field imaging used in this work, immobilization of 2–3 minutes is sufficient for localizing a worm of interest and taking a picture. For confocal imaging, the time range required to run through a *z*-stack is in the range of ~1 min up to 5–6 minutes (depending on the depth of the scan and the averaging number). For this operation range (*i*.*e*. immobilization time ≤ 10 min, 30 or 40 μm beads), we did not observe a statistically significant decrease of the thrashing frequency after worm recovery. We conclude that immobilization conditions with our method are suitable for common imaging protocols. On the other hand, as shown in Fig 4K, tetramisole-treated worms did not recover from anesthesia after 10 min. Based on our observations, it takes 2 to 4 hours for the 1mM tetramisole-treated worms to recover to about 80% of the thrashing frequency level of the control. To evaluate possible permanent immobilization effects, the thrashing frequency was also measured 1 day after release, to leave enough time for tetramisole-treated worms to recover. The results in Fig 4K show that in this case after short-term immobilization the thrashing frequency decreases by about 30% compared to control and to gel immobilization, indicating an adverse effect on their physical state. This effect is enhanced after long-term immobilization. For the extreme case of maximum compression using the gel-based matrix (60 min, 30 μm beads), a reduction of only about 20% in the thrashing frequency of the released worms was observed, indicating still a reasonably good physiological state of the worms.

In the fertility assay, we measured the number of progeny produced per worm. This analysis was done 1, 2 or 3 days after recovery from immobilization. Worms were recovered from 1 hour or 2 hours of gel immobilization with 30 μm beads. We compared these numbers with a tetramisole-treated population (1 hour) and a control population, respectively. In Fig 4L, we observed a general decrease in fertility with increasing age for all groups. Furthermore, an about 30% of fertility decline at day 1 and day 2 of tetramisole-treated worms compared to the control group, which indicates an adverse effect of this method, possibly related to muscle paralysis in the reproductive system caused by the anesthetic. For our hydrogel-microbead immobilization method, we do not observe a significant decline with respect to the control group, indicating no obvious adverse physiological effects.

### High-resolution confocal fluorescent imaging of mitochondrial networks in *C*. *elegans* using different immobilization techniques

**Fig 5:**
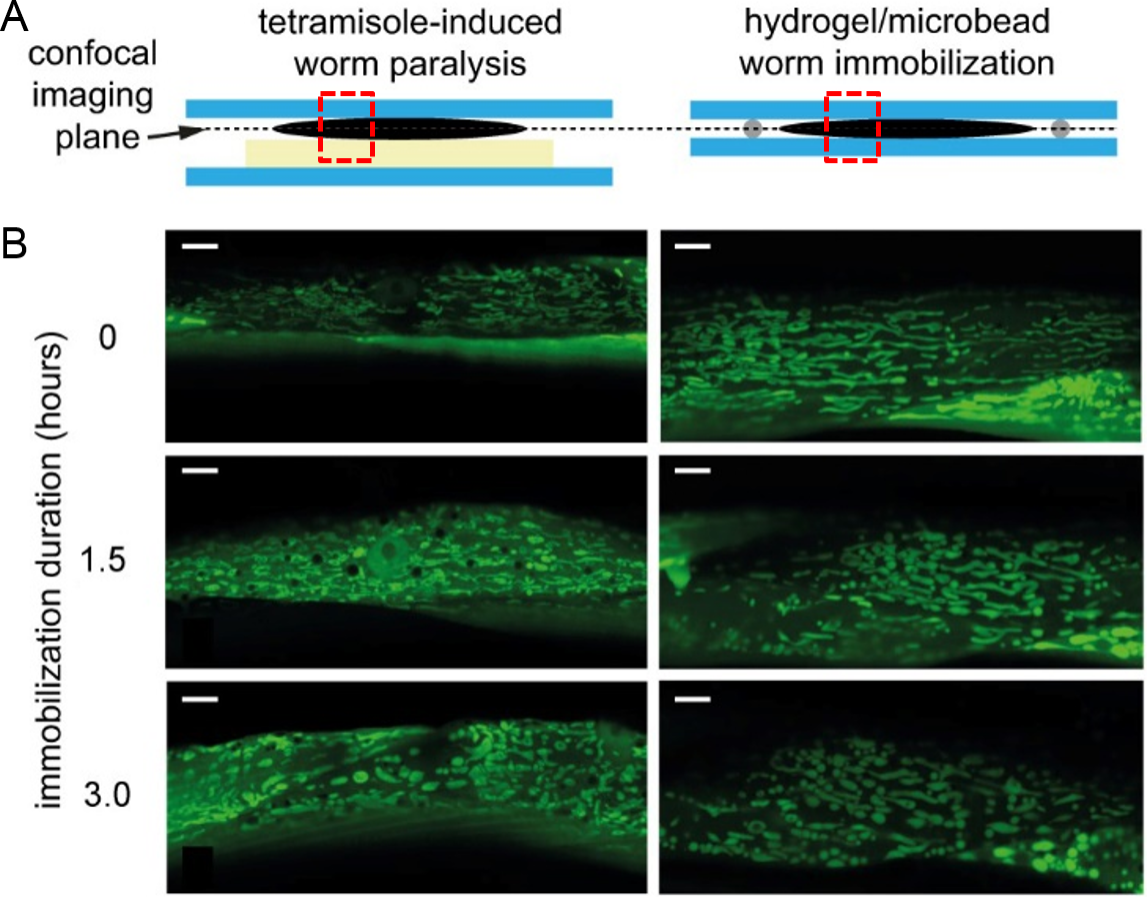
High-resolution confocal imaging of *C*. *elegans*. (A) Schematics of an immobilized adult worm (side view) using two different techniques, *i*.*e*. tetramisole-induced worm paralysis (current gold standard) and our gel-based method (dashed rectangles indicate the location of the imaging areas). (B) Representative confocal fluorescent images of dynamic alterations of mitochondrial networks in *C*. *elegans* body wall muscle cells during continuous immobilization up to 3 hours, visualized using the *Pmyo-3*::*mito*::*GFP* reporter. Images are taken through a 63× NA 1.4 oil immersion objective using the standard immobilization method (left panels) and our technique (right panels). Scale bars = 5 μm.

Prior to the imaging of protein aggregation in neurodegenerative worm strains by wide-field fluorescent microscopy, we evaluated our gel-based immobilization technique through the challenging application of confocal imaging of mitochondrial morphology, which has also been associated with several neurodegenerative disorders [6]. Using a transgenic strain expressing the GFP in mitochondria under the control of the muscle-specific *myo-3* promoter (*Pmyo-3*::*mito*::*GFP* reporter), we compared the quality of the acquired images obtained with the gel-based technique and with the traditional anesthetics-based immobilization using tetramisole. Fig 5A schematically compares the two techniques. Here, as in the following experiments, PF127 with 30 μm spacer microbeads was used for immobilization. For this evaluation, worms have been immobilized continuously during 3 hours. Thanks to very stable worm immobilization provided by both techniques, the features of interest could be imaged without re-adjusting the objective position over the entire observation time. Fig 5B presents high-resolution confocal fluorescent pictures of mitochondrial networks during 3 hours immobilization. For both methods, mitochondrial networks maintained an organized interconnected morphology over 1.5 hours and started displaying signs of fragmented pre-apoptotic mitochondria after ~3 hours of immobilization. Both techniques show a similar degeneration of the mitochondrial network over time, indicating that this evolution is actually not merely a side effect of anesthesia. The observed alteration of the network is probably due to different stress factors experienced by the worm, *e*.*g*. a lack of food or oxygen deprivation with both methods during this long immobilization interval. Anoxic and hypoxic conditions are known to induce mitochondrial fission [40]. Such harsh conditions are not an issue for imaging experiments discussed in this work, which, as mentioned earlier, require maximum continuous immobilization intervals lasting not more than ~10 min. As outlined above, the major advantage of the PF127 immobilization protocol is its reversibility, thus opening the possibility of longitudinal studies over several days (see below). Furthermore, the anesthetics-based technique requires extra preparatory steps, *e*.*g*. for the creation of an agar pad. Also, agar is not a good optical medium due to its yellowish translucent coloring, whereas PF127 gel is perfectly transparent, thus improving the imaging quality. Due to the slight compression of worms with our technique, a slightly larger portion of the worm muscle cells are located within the imaging focal plane, allowing for a more extensive visualization of the mitochondrial network.

### Mitochondrial morphology progression during worm aging and for different RNAi conditions

As a proof-of-concept for performing longitudinal studies based on high-resolution confocal imaging of *C*. *elegans*, we investigated mitochondrial morphology in the body wall muscle cells of worms under different RNAi conditions and at different timing during their early adult life. In mammals, mitochondrial fission is mediated by a single protein called DRP1, whereas fusion requires two families of proteins, MFN1/MFN2 and OPA1 [41]. Mitofusins MFN1/MFN2, or *C*. *elegans* homologue *fzo-1*, are required for the mitochondrial outer membrane fusion [42] and OPA1, or *C*. *elegans* homologue *eat-3*, is required for the inner membrane fusion [43]. *fzo-1* depleted worms are known to display a fragmented and disorganized mitochondrial network morphology [44]. On the other hand, *drp-1* deficient worm’s display disorganized aggregated and globular mitochondria that can be visualized as large blebs [45]. The mitochondrial network in wild-type animals is well-organized and highly interconnected [44].

**Fig 6:**
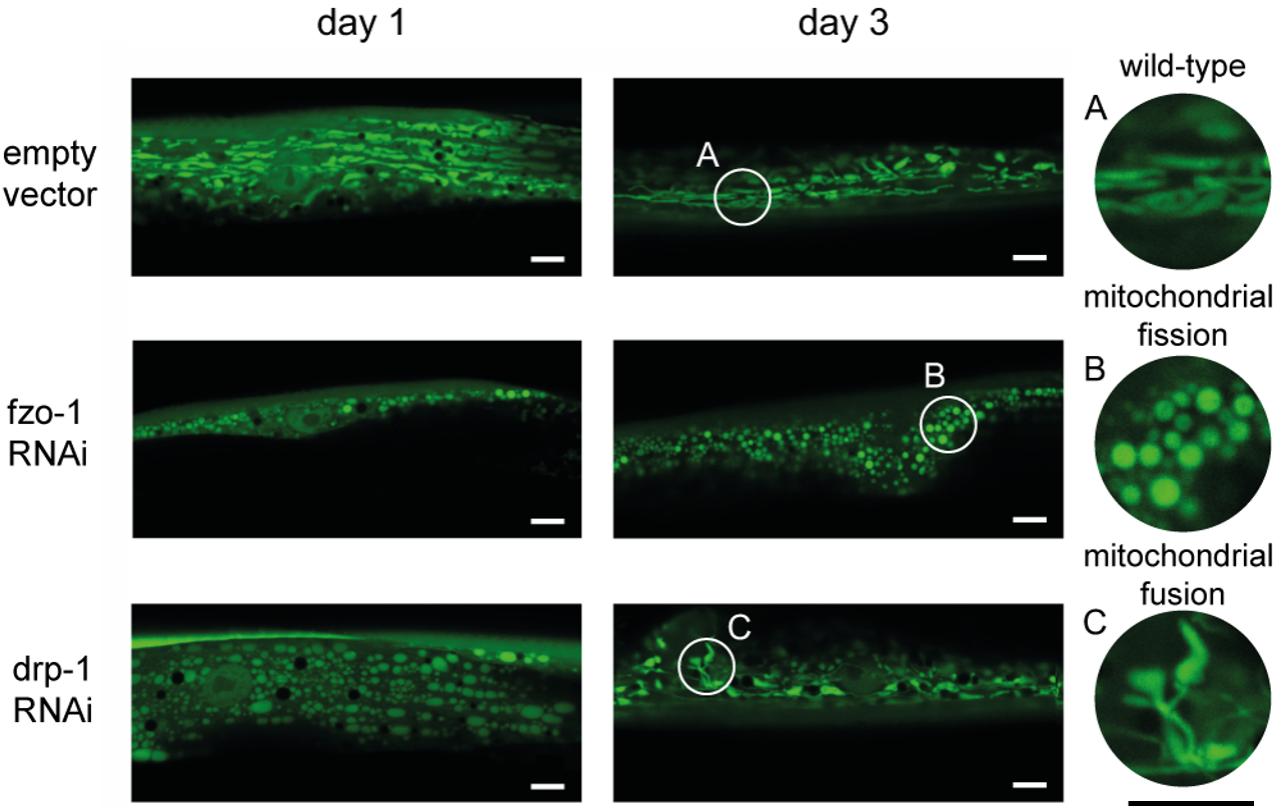
Longitudinal analysis of mitochondrial morphology in the body wall muscle cells of *C*. *elegans* worms under different RNAi conditions. In wild-type control worms (empty vector), the mitochondrial network is well interconnected on day 1, but begins to fragment with age on day 3 (inset A). *fzo-1* knockdown inhibits mitochondrial fusion and produces many fragmented mitochondria patterns (fission, inset B), whereas *drp-1* RNAi knockdown inhibits division and produces larger aggregated networks (fusion, inset C). Images are representative of *n* = 4–6 worms analyzed per condition, visualized via confocal microscopy (63× NA 1.4 oil immersion objective) using the *Pmyo-3*::*mito*::*GFP* reporter. Scale bars = 5 μm. Insets A, B and C are digital zooms (x3.6) of the indicated circular areas.

These observations were confirmed using the gel-based imaging technique, with 63× confocal imaging of the mitochondrial structures in the body wall muscle cells of *C*. *elegans* (Fig 6). For this study, worms were immobilized in the PF127-microbead matrix for imaging at day 1 and day 3 of adulthood, and allowed to grow on agar plates in the interval. In control worms, treated with an empty RNAi vector, the mitochondrial network was well interconnected on day 1 (young adult stage), but began to fragment with age on day 3 (Fig 6, Inset A). During cell death, it is known that mitochondria dramatically fragment as a consequence of an increased recruitment of *drp-1* to mitochondria [46]. *fzo-1* RNAi knockdown inhibited mitochondrial fusion and produced many fragmented mitochondria (Fig 6, Inset B), whereas *drp-1* RNAi knockdown inhibited division and produced larger aggregated mitochondria (Fig 6, Inset C). There was a certain degree of heterogeneity between different muscle cells of the same worm, probably pointing to the limited efficiency of the RNAi treatment (images not shown). The digital zooms shown as insets in Fig 6 reveal submicrometer-size features, emphasizing the stability of worm immobilization with our technique. S3 Movie shows a time-lapse video of a fluorescent mitochondrial network in the body wall muscles of an immobilized worm, where mitochondrial fusion could be observed.

### High-resolution imaging of progressing protein aggregation in different *C*. *elegans* neurodegenerative disease models

**Fig 7:**
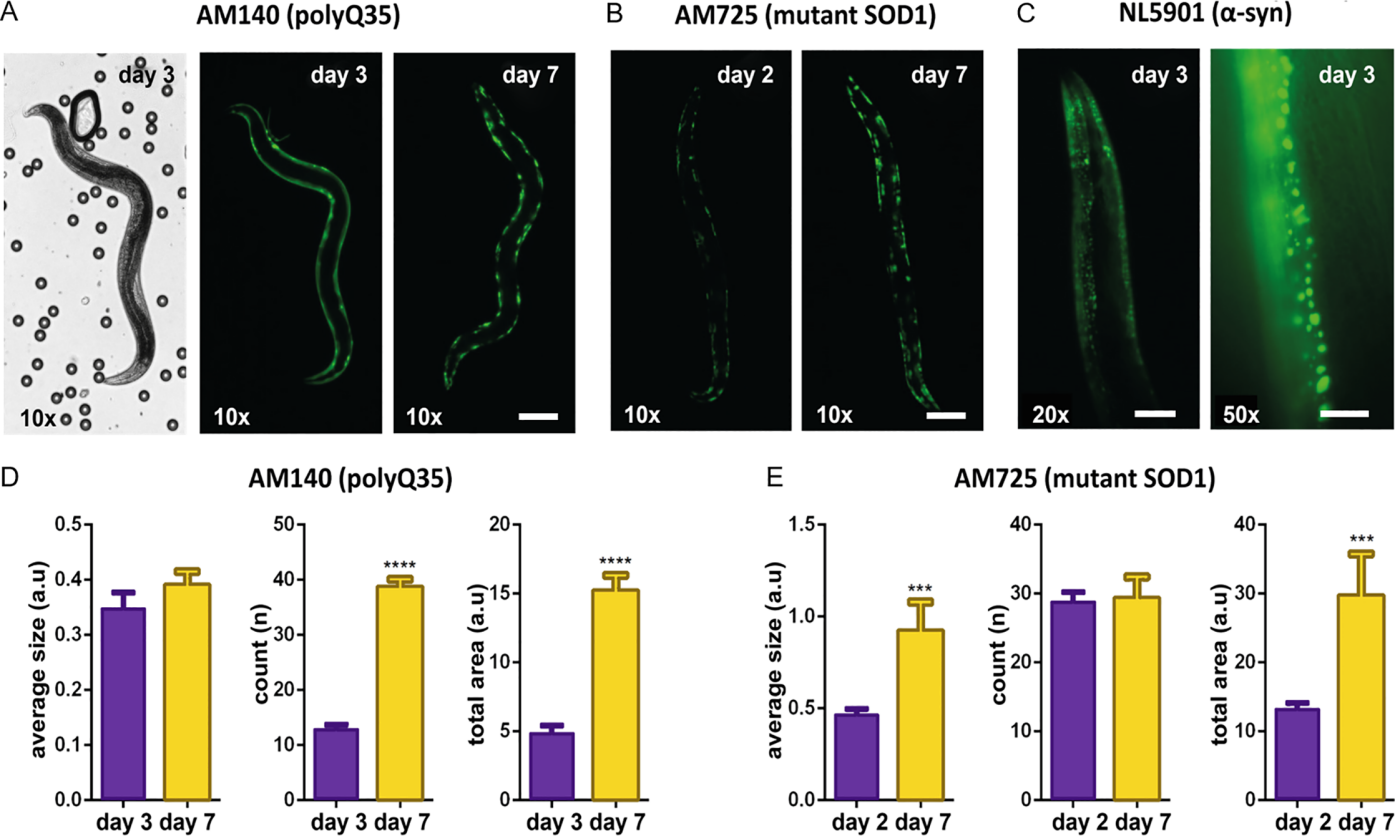
Protein aggregate morphology and progression in different *C*. *elegans* neurodegenerative disease models. (A) Time-lapse bright field and fluorescent wide field images of an AM140 worm (Huntington’s disease model) immobilized in the gel-microbead matrix. PolyQ35 proteins exhibit a transition from a soluble state to an aggregated form as the worm ages from day 3 to day 7 of adulthood (10× objective, scale bar = 100 μm). (B) Time-lapse fluorescent pictures of an AM725 worm (Amyotrophic Lateral Sclerosis model) displaying aggregated SOD1 proteins throughout its adulthood (day 2 to day 7), with aggregate size growing over time (10× objective, scale bar = 100 μm). (C) Fluorescent images of α-syn aggregates in a NL5901 worm (Parkinson’s disease model), allowing accurate visualization of smaller aggregates (20× and 50× objectives, scale bars = 50 μm and 20 μm, respectively). (D-E) Temporal evolution (from day 2 or 3 to day 7) of the average aggregate size, number of aggregates and total aggregate area per worm, in (D) AM140 and (E) AM725 worms. Data is presented as mean+SEM, ***p≤0.001 and ****p≤0.0001, *n* = 15–20 for each group).

Protein aggregation in *C*. *elegans* models of Huntington’s disease (HD), Parkinson’s disease (PD), and Amyotrophic Lateral Sclerosis (ALS) was characterized by wide-field high-resolution fluorescent imaging of distinct proteins within the worm muscular tissues. In particular, for HD the AM140 transgenic strain was used, showing Q35::YFP expression in the body wall muscles [47]. For ALS, we employed the AM725 strain, encoding a mutated form of human SOD1::YFP expressed in body wall muscle cells [48]. PD was modeled using the NL5901 strain, featuring α-syn::YFP expression in body wall muscles [49]. Aggregate morphology and accumulation were observed using the described gel-based technique. Each disease model was found to display different aggregate morphologies and evolution with aging. For HD, the AM140 strain was chosen for imaging due to easier visualization of aggregates in the large body wall muscle cells. In this model, polyglutamine (polyQ) repeats progressively underwent a transition from a soluble non-aggregated form to the insoluble aggregate formation with age (Fig 7A) [50]. In contrast, ALS worms with mutant SOD1 aggregation displayed aggregate formation right from the embryo stage (figures not shown), pointing to the permanent instability of the protein. Disease progression, in this case, is more due to the increased expression and accumulation of mutated SOD1 in the worm’s body wall muscle cells (Fig 7B). In the case of PD, α-syn aggregates were difficult to visualize at full-worm scale (*i*.*e*. through a 10× microscope objective), due to their much smaller size with respect to the largely elongated foci formed by polyQ repeats and SOD1 aggregates. Nevertheless, the high stability performance offered by our worm immobilization method allowed obtaining clear images of small α-syn aggregates as well, by using higher magnification objectives (20× and 50× in Fig 7C). In this case, aggregate counting and statistical size quantification per worm would be hence possible by stitching multiple pictures to cover the entire worm body.

For the HD and ALS worm models, quantitative information related to the average size, number and total area of aggregates could be extracted over multiple days through basic image processing. The total aggregate area is not an independent parameter but the product of the number and the average size of aggregates. Interestingly, in the HD model, the number of aggregates increased significantly with aging, but with the aggregate size remaining nearly constant (Fig 7D). For the ALS model, however, this effect was reversed and the aggregate size was the main indicator of disease progression (Fig 7E).

### Immobilization of other small model organisms

**Fig 8:**
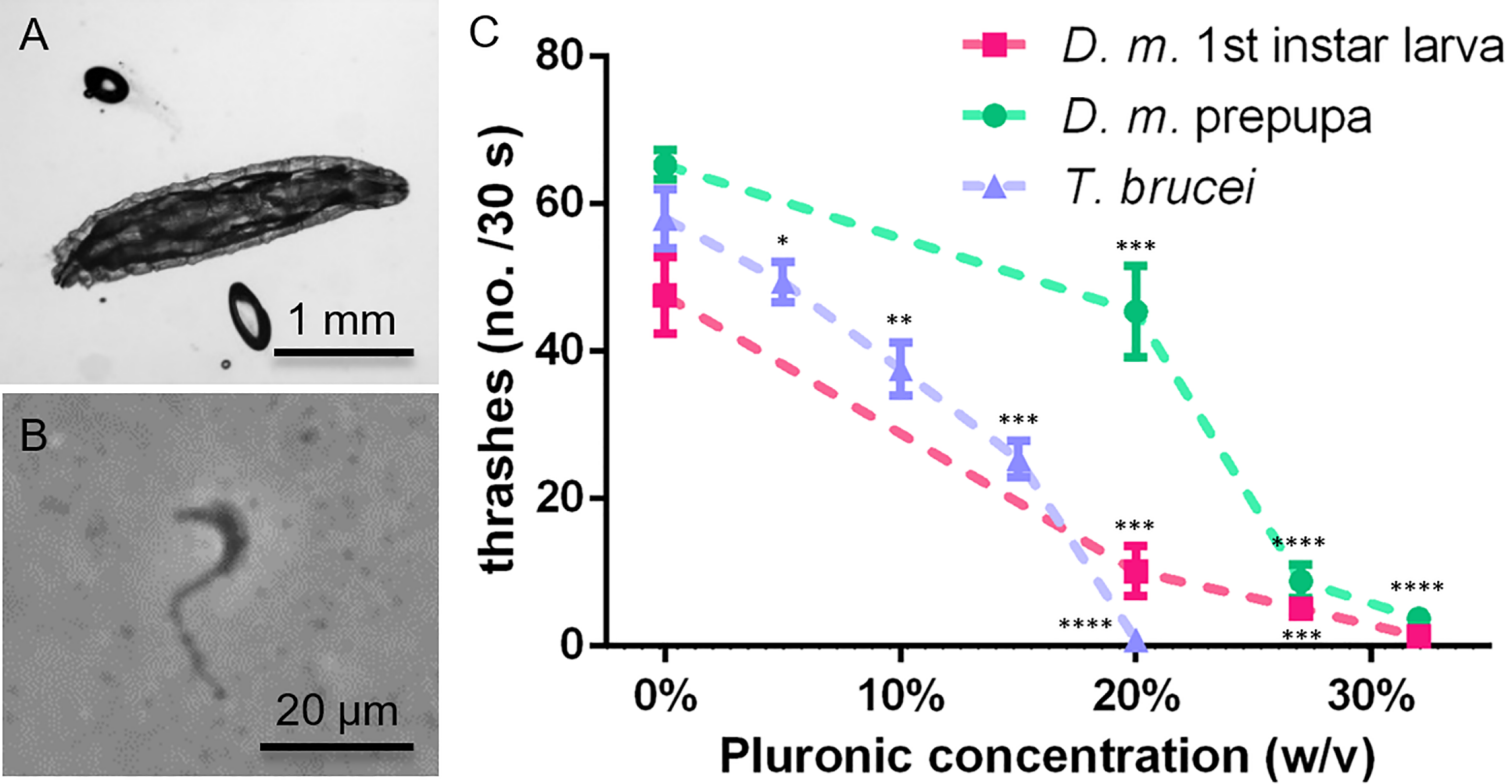
Gel-based immobilization of other small organisms. (A) Image of an immobilized *D*. *melanogaster* 1^st^ instar larva (length ~2 mm). (B) Image of an immobilized *T*. *brucei* unicellular parasite (length ~20 μm). (C) Pluronic hydrogel concentration-related reduction of head thrashes for *D*. *melanogaster* 1^st^ instar larvae, *D*. *melanogaster* larvae in the prepupal stage (n = 15 for each group), and reduction of body thrashes (swimming frequency) for *T*. *brucei* (n = 20). Data is present as mean±SD, * p≤0.05, ** p≤0.01, *** p≤0.001 and **** p≤0.0001 *vs* each 0% w/v Pluronic group, n = 15–20 for each group.

Finally, to test the versatility of our immobilization protocol, we applied it to other small biological model organisms, such as *D*. *melanogaster* larvae and *T*. *brucei* unicellular parasites. Both these organisms could actually be immobilized in PF127 (30% w/v) hydrogel only (no microbeads were required). In the case of *D*. *melanogaster*, this is somewhat surprising, as *D*. *melanogaster* larvae are much larger (length ~2 mm in the first instar stage, <4 mm in the prepupal stage), and possibly develop stronger locomotion forces than *C*. *elegans* adult worms (length ~1 mm) (Fig 8A). However, due to the larger body size, they are also subjected to higher viscous forces. Importantly, there is also a major difference in the natural body motion pattern, which is composed of lateral undulations in the case of *C*. *elegans* worms, whereas *D*. *melanogaster* larvae locomotion is induced by circular muscles squeezing and extending the larval body along its axis. In order to evaluate the effect of the PF127 concentration, in this case, we measured the frequency of the small lateral head thrashes and found that for Pluronic concentrations ≥27% w/v *D*. *melanogaster* larvae can be well immobilized (Fig 8C). On the other hand, *T*. *brucei* is a very small undulating organism (length 20 to 30 μm) that cannot generate forces strong enough, similar to small *C*. *elegans* larvae, to move in highly viscous Pluronic gels (Fig 8B). In this case, we evaluated, as for *C*. *elegans*, the frequency of locomotory body thrashes and found that for a PF127 concentration of 20% w/v *T*. *brucei* becomes immobile (Fig 8C).

## Conclusion

We reported a new and simple method for the immobilization of *C*. *elegans* nematodes, based on a biocompatible and temperature-responsive hydrogel-microbead matrix. Worms were confined in a thermogelling Pluronic (PF127) droplet and clamped in between two glass slides, thus the method does not rely on a specific chip design, nor does it require a complicated device fabrication process. Microbeads (Ø 30 μm) mixed with the gel functioned as spacers for our imaging experiments, providing well-controlled immobilization parameters, thereby avoiding adverse effects on worms’ physiology due to excessive compression. A PF127 concentration of 30% w/v in buffer solution was found to offer best operation parameters, *i*.*e*. high viscosity at room temperature and a suitable sol-gel transition temperature (~12°C). The combination of viscoelastic and friction forces enabled reliable short-term or long-term (> 1 hour) immobilization of *C*. *elegans*. The technique allows recovery of the worms, *e*.*g*. for repeated imaging of the same worm over a time span of several days, a feature that presents a significant advantage with respect to conventional anesthetics- or glue-based methods. Furthermore, in pluronic only, immobilized animals often rotate around their longitudinal axis, hence preventing stable observation and imaging of features in their body. This happens in particular during fluorescence imaging, since such movement can be triggered by the worm’s propensity to avoid blue light. As with our method the worm is slightly compressed, rotation around its axis can not take place.

To test the performance of our technique, we focused on biological applications requiring challenging longitudinal high-resolution imaging and data quantification related to the morphology, size and/or counting of specific shapes within the worms’ tissues. In particular, we demonstrated high-quality *in vivo* imaging of mitochondrial morphology in the body wall muscle cells of *C*. *elegans* by confocal fluorescent microscopy on immobilized worms. This allowed successfully observing the long-term dynamics of the mitochondrial fusion and fission processes with unprecedented simplicity and accuracy. Likewise, the progression of protein aggregates, one of the major hallmarks of many neurodegenerative diseases, was assessed quantitatively in different *C*. *elegans* models of human neurodegenerative diseases, namely ALS, HD, and PD. In this study, our method enabled the longitudinal quantification of size and number of specific protein aggregates in each worm, revealing clear differences in the temporal evolution of the protein aggregation patterns in HD and ALS, while allowing the observation of particularly small aggregates, via high-magnification imaging of PD worm models. Finally, the hydrogel-based technique was also successfully applied to other biological model organisms, such as *D*. *melanogaster* larvae and *T*. *brucei* parasites.

With our approach, immobilization of small organisms in the precooled Pluronic gel is achieved by natural thermalization of the glass/PF127 substrate at room temperature, thus no specific equipment or technical skills are required, making the technique readily operational in any biological laboratory. Because of its high performance, versatility and ease of use, we believe that our immobilization technique represents a valid alternative to currently used practice with high potential to improve the quality of biological assays and longitudinal studies based on long-term observation and imaging of single worms or small worm populations.

## Supporting information

S1 Movie Head region. Time-lapse video (duration 34 minutes, 5 s intervals) of the mitochondrial network in the head region (pharynx) of a gel-immobilized *myo-3* worm with 30 μm microbeads (63×, fluorescence imaging).
(MP4)

S2 Movie Mid-region. Time-lapse video (duration 30 minutes, 5 s intervals) of the mitochondrial network in the mid-region (near vulva) of a gel-immobilized *myo-3* worm with 30 μm microbeads (63×, fluorescence imaging).
(MP4)

S3 Movie Mitochondrial fusion. Time-lapse video (duration 30 minutes, 5 s intervals) of mitochondrial fusion processes in a body wall muscle between head and mid-region of a gel-immobilized *myo-3* worm with 30 μm microbeads (63×, fluorescence imaging).
(MP4)
